# In the light of deep coalescence: revisiting trees within networks

**DOI:** 10.1186/s12859-016-1269-1

**Published:** 2016-11-11

**Authors:** Jiafan Zhu, Yun Yu, Luay Nakhleh

**Affiliations:** 1 Department of Computer Science, Rice University, Houston, 77005 Texas USA; 2 Department of BioSciences, Rice University, Houston, 77005 Texas USA

## Abstract

**Background:**

Phylogenetic networks model reticulate evolutionary histories. The last two decades have seen an increased interest in establishing mathematical results and developing computational methods for inferring and analyzing these networks. A salient concept underlying a great majority of these developments has been the notion that a network displays a set of trees and those trees can be used to infer, analyze, and study the network.

**Results:**

In this paper, we show that in the presence of coalescence effects, the set of displayed trees is not sufficient to capture the network. We formally define the set of parental trees of a network and make three contributions based on this definition. First, we extend the notion of anomaly zone to phylogenetic networks and report on anomaly results for different networks. Second, we demonstrate how coalescence events could negatively affect the ability to infer a species tree that could be augmented into the correct network. Third, we demonstrate how a phylogenetic network can be viewed as a mixture model that lends itself to a novel inference approach via gene tree clustering.

**Conclusions:**

Our results demonstrate the limitations of focusing on the set of trees displayed by a network when analyzing and inferring the network. Our findings can form the basis for achieving higher accuracy when inferring phylogenetic networks and open up new venues for research in this area, including new problem formulations based on the notion of a network’s parental trees.

## Background

**Fig. 1:**
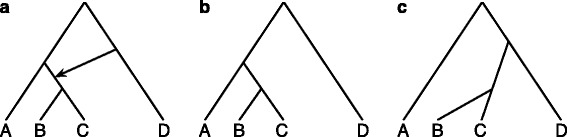
**a** A phylogenetic network *ψ* with one reticulation node and (**b-c**) the two trees it displays

Evolutionary, or explicit, phylogenetic networks are graphical models that model reticulate evolutionary histories [1–3]. Such evolutionary histories arise when processes such as horizontal gene transfer or hybridization occur. Research into mathematical properties, complexity results, and algorithmic techniques has exploded recently, as evident by the publication of three recent books on the subject [4–6]. A main premise behind the use of phylogenetic networks is that when a single tree is not sufficient to model the evolutionary history of a set of sequences or characters, a phylogenetic network that encompasses several trees is used. For example, the phylogenetic network in Fig. 1 a depicts an evolutionary history that involves hybridization between taxon D and the most recent common ancestor (MRCA) of taxa B and C.

Central to research on phylogenetic networks has been the notion of trees *displayed* by a phylogenetic network. We say that a phylogenetic network displays a tree if the tree can be obtained by removing a set of “reticulation edges” of the network. Figure 1 shows the two trees displayed by the network given in the figure. Given a phylogenetic network *ψ*, we denote by $\mathcal {U}(\psi)$ the set of all trees displayed by *ψ*. When incongruence in the gene trees inferred on different genomic regions across a genome alignment is assumed to be caused only by reticulation (e.g., hybridization), then the observed gene trees are taken to be a subset of the set of trees displayed by the (unknown) phylogenetic network for the set of genomes. This is why the set $\mathcal {U}(\psi)$ has played a fundamental role in most results established for phylogenetic networks. Examples of the prominent use of $\mathcal {U}(\psi)$ include: (1) Parsimonious phylogenetic networks that fit the evolution of a set of sequences under the infinite sites model [7–14]; (2) extending the maximum parsimony and maximum likelihood criteria from trees to networks [15–20]; (3) inferring minimal networks from sets of gene trees [21–24]; (4) establishing identifiability results related to networks [25]; (5) establishing complexity results related to networks [26–31]; and (6) identifying special trees within the network [32–35].

One of the evolutionary phenomena that has been extensively documented in recent analyses and targeted for computational developments is *deep coalescence*, or *incomplete lineage sorting* [36]. This phenomenon amounts to gene tree incongruence due to population effects (determined by factors such as the sizes of ancestral populations and/or the times between subsequent speciation events). When this phenomenon is present in a reticulate evolutionary history, a major challenge faces all the aforementioned works: The set of trees displayed by a network is no longer adequate to fully capture gene evolution within the network.

To resolve this issue, we define the set of parental trees of a phylogenetic network to supplant the set of displayed trees (this is the same as the set of *weakly displayed trees* defined by Huber et al. [37]). Based on this set, we make three contributions. First, we extend the concept of anomaly zone to phylogenetic networks and establish results based on this concept. It is important to note here that Solís-Lemus et al. [38] recently discussed the issue of anomaly in the presence of reticulation where they focused on the “species tree” inside the network. Here, we define the anomaly zone in terms of the set of all parental trees and do not designate a species tree inside the network. Second, we address the problem of inferring a backbone tree inside the network that could serve as a starting tree for network searches and/or provide information on the history of speciation events in the presence of gene flow. As in the first contribution, the work here differs from that of [38] in focusing on all trees displayed by a network, rather than just a designated species tree. Third, we propose a novel clustering-based approach to phylogenetic network inference from gene trees by which the gene trees are first clustered, parental trees are inferred from the clusters, and then the parental trees are combined into a phylogenetic network. Gori et al. [39] recently studied the performance of various combinations of dissimilarity measures and clustering techniques in clustering gene trees. Our work differs from that of [39] in that our focus is on phylogenetic network inference via clustering.

We believe our work will open up new venues for research into computational methods and mathematical results for reticulate evolutionary histories.

## Methods

We focus here on binary evolutionary (or, explicit) phylogenetic networks [2].

### **Definition 1**

The topology of a phylogenetic network *ψ* is a rooted directed acyclic graph (*V*,*E*) such that *V* contains a unique node with in-degree 0 and out-degree 2 (the root) and each of the other nodes has either in-degree 1 and out-degree 2 (an internal tree node), an in-degree 1 and out-degree 0 (an external tree node, or leaf), or in-degree 2 and out-degree 1 (a reticulation node). The leaves are bijectively labeled by a set of taxa. The phylogenetic network has branch lengths *λ*, such that *λ*_*b*_ denotes the length of branch *b* in *ψ* in coalescent units.

As we discussed in the Background section and illustrated in Fig. 1, the notion of trees displayed by a network has played a central role in analyzing and inferring networks.

### **Definition 2**

Let *ψ* be a phylogenetic network. A tree *t* is displayed by *ψ* if it can be obtained by removing for each reticulation node exactly one of the edges incident into it followed by repeatedly applying forced contractions until no nodes of in- and out-degree 1 remain. A forced contraction of a node *u* of in-degree 1 and out-degree 1 consists of (1) adding an edge from *u*’s parent to *u*’s child, and (2) deleting node *u* and the two edges that connect it to its parent and child. We denote by $\mathcal {U}(\psi)$ the set of all trees displayed by *ψ*.

Figure 1 shows a phylogenetic network *ψ* along with $\mathcal {U}(\psi)$.

### Deep coalescence and the parental trees inside a network

Let us consider tracing the evolution of a recombination-free genomic region of four individuals *a*,*b*,*c*, and *d*, sampled from the four taxa A, B, C and D within the branches of the phylogenetic network *ψ* of Fig. 1. If *b* and *c* coalesce at the most recent common ancestor (MRCA) of B and C, and no events such as deep coalescence or duplication/loss occur anywhere in the phylogenetic network, then the genealogy of the genomic region is one of the two trees in the set $\mathcal {U}(\psi)$. This is precisely the reason why much attention has been given to the set $\mathcal {U}(\psi)$, as discussed in the Background section.

**Fig. 2:**
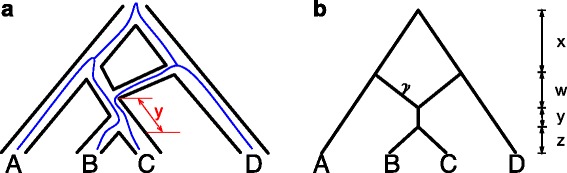
**a** The genealogy of a gene (*blue* lines) within the branches of a phylogenetic network. In this scenario, the two lineages from B and C failed to coalesce prior to the reticulation node (evolution is viewed backward in time, from the leaves toward the root). The resulting genealogy in this case is ((a,b),(c,d)) and neither of the two trees in the set $\mathcal {U}(\psi)$ (shown in Fig. 1) capture this scenario. The length in coalescent units of the branch between the reticulation node and the MRCA of B and C is *y*. **b** An abstract representation of the network, assuming both reticulation edges have the same length *w*



However, let us now consider a scenario where *b* and *c* did not coalesce at the MRCA of B and C. One potential outcome in terms of the resulting genealogy for *a*,*b*,*c*, and *d* is illustrated in Fig. 2 a. The probability that *b* and *c* fail to coalesce at the MRCA of B and C has to do with the quantity *y* in the figure: The smaller it is, the more likely it is that *b* and *c* would fail to coalesce [40]. Interestingly, for the scenario illustrated in Fig. 2 a, neither of the two trees in the set $\mathcal {U}(\psi)$ can capture the shown genealogy. This brings us to define the set of parental trees inside a phylogenetic network to appropriately represent the network as a mixture of trees that adequately model the evolution of genes in the presence of deep coalescence. Parental trees are what Huber et al. referred to as *weakly displayed trees* in [37].

**Fig. 3:**
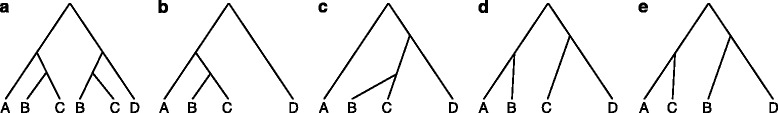
**a** The MUL-tree of the phylogenetic network *ψ* in Fig. 1a. **b**–**e** The four trees that constitute set $\mathcal {W}(\psi)$, assuming one individual is sampled per species

Yu et al. [41] gave an algorithm for the simple task of converting a phylogenetic network *ψ* to a multi-labeled tree, or MUL-tree, *T*. Proceeding from the leaves of the network toward the root, the algorithm creates two copies of each subtree rooted at a reticulation node, attaches them to the two parents of the reticulation node, and deletes the two reticulation edges. See Fig. 3 a for an illustration. Notice that multiple leaves could be labeled with the same taxon name, and hence the MUL-tree naming. The pseudo-code for converting a phylogenetic network into a MUL-tree is given in Algorithm 1, where *T*_*w*_ denotes the subtree of tree *T* rooted at node *w* (node *w* and all the nodes and edges “under” it in the tree).

As phylogenomic analyses are increasingly involving multiple individuals per species, we provide a general definition of parental trees that applies to cases with multiple individuals per species. Let $\mathcal {X}$ be the set of species and *a*_*x*_ denote the number of genomes sampled from species $x \in \mathcal {X}$. Let *T* be a MUL-tree. We denote by $T|_{(\mathcal {X},a)}$ a tree obtained from *T* by retaining, for each taxon $x \in \mathcal {X}, n_{x}$ leaves labeled by *x*, where 1≤*n*_*x*_≤*a*_*x*_, and deleting the remaining (*a*_*x*_−*n*_*x*_) leaves labeled by *x*, followed by repeatedly applying forced contractions until no nodes of in- and out-degree 1 remain.

#### **Definition 3**

Let *ψ* be a phylogenetic network on set $\mathcal {X}$ of taxa and *T* be its MUL-tree. A parental tree inside *ψ* is a tree *t* such that $t=T|_{(\mathcal {X},a)}$. We denote by $\mathcal {W}(\psi)$ the set of all parental trees inside *ψ*.

Figure 3 shows the set $\mathcal {W}(\psi)$ for the phylogenetic network in Fig. 1. The gene genealogy shown in Fig. 2 a can be captured by the parental tree in Fig. 3 d. Indeed, Yu et al. [41, 42] gave mass and density functions for gene trees on phylogenetic networks in terms of the set of parental trees inside the network. While it is obvious that $\mathcal {U}(\psi) \subseteq \mathcal {W}(\psi)$, the two sets can differ significantly in terms of their properties. For example, if *ψ* has *k* reticulation nodes, then $|\mathcal {U}(\psi)| \leq 2^{k}$. However, $|\mathcal {W}(\psi)|$ could be much larger than 2^*k*^, as it is a function of the numbers of leaves under the reticulation nodes as well as the numbers of individuals sampled per species.

One rather interesting result is that while the problem of testing whether a tree is displayed by a network is NP-hard [28], testing whether a tree is a parental tree of (equivalently, weakly displayed by) a network can be done in polynomial time [37].

### Inheritance probabilities and the multispecies network coalescent

Given a species tree topology *ψ* and its branch lengths *λ*, the gene tree topology *G* can be viewed as a discrete random variable whose mass function *P*_*ψ*,*λ*_(*G*=*g*) was derived in [40]. In the case of phylogenetic networks, we also associate with every pair of edges *b*_1_=(*u*_1_,*v*) and *b*_2_=(*u*_2_,*v*) that are incident into the same reticulation node *v* nonnegative real values $\gamma _{b_{1}}$ and $\gamma _{b_{2}}$ such that $\gamma _{b_{1}}+\gamma _{b_{2}}=1$ [41, 42]. These quantities, which we call inheritance probabilities, indicate the proportions of lineages in hybrid populations that tracks each of the two parents of that population. In this case, the phylogenetic network’s topology *ψ* and branch lengths *λ*, along with the vector of inheritance probabilities *Γ*, are sufficient to describe the mass function of gene trees *P*_*ψ*,*λ*,*Γ*_(*G*=*g*) under the multispecies network coalescent [41, 42].

## Results and discussion

In this section we describe the three main contributions of this work. First, we extend the concept of anomaly zones [43] to phylogenetic networks and establish conditions for their existence. Second, we address the question of whether it is possible, from an inference perspective, to obtain a tree that can be augmented into the correct network by adding reticulation edges between pairs of the tree’s edges. Third, we propose a clustering approach to network inference by clustering the gene trees, inferring parental trees, and then combining the parental trees into a network. These results have direct implications not only on understanding the relationships between trees and networks, but also the practical task of developing computational methods for network inference.

### Phylogenetic networks and anomalies

In a seminal paper, Degnan and Rosenberg [43] showed that the branch lengths of a species tree could be set such that the most likely gene tree disagrees with the species tree. Such a gene tree is called an *anomalous gene tree* and the set of all branch length settings that result in an anomalous gene tree is the *anomaly zone*.

We now provide what, to the best of our knowledge, is the first definition of anomaly zones for phylogenetic networks. Note that in [38], Solís-Lemus et al. discussed anomalous gene trees in the presence of ILS and gene flow. However, in their work, the anomaly was still defined with respect to a designated species tree (they viewed the phylogenetic network as a species tree with additional horizontal edges between pairs of its branches). Here, we do not designate any of the parental trees of the network as a species tree; instead, we define the anomaly zone directly in terms of the entire set of parental trees.

The guiding principle behind our definition is the question: Is the most likely gene tree to be generated by a phylogenetic network necessarily a parental tree inside the network?

#### **Definition 4**

Let *ψ*be a phylogenetic network, *λ* be its branch lengths, and *Γ* be the inheritance probabilities associated with its reticulation edges. We say gene tree topology *g* is anomalous for (*ψ*,*λ*,*Γ*) if

1 $$  P_{\psi,\lambda,\Gamma}(G=g)>P_{\psi,\lambda,\Gamma}(G=t) ~\forall{t \in \mathcal{W}}(\psi).  $$

A phylogenetic network *ψ* is said to produce anomalies if there exists branch lengths *λ* and inheritance probabilities *Γ* such that there exists an anomalous gene tree *g* for (*ψ*,*λ*,*Γ*). The anomaly zone for a phylogenetic network *ψ* is a set of (*Λ*,*Γ*) values for which *ψ* produces anomalies.

Degnan and Rosenberg [43] showed that three-taxon and symmetric four-taxon species trees have no anomaly zones, but that non-symmetric four-taxon trees and all species trees with five or more taxa have anomaly zones. One practical implication of these results was that the simple approach of sampling a very large number of loci, building gene trees and taking the most frequent gene tree as the species tree (an approach dubbed “the democratic vote” method) does not always work.

Since the multispecies coalescent is a special case of the multispecies network coalescent, it immediately follows that any phylogenetic network with *n*≥5 leaves produces anomalies. We now show that three-taxon phylogenetic networks do not produce anomalies, but that symmetric phylogenetic networks with *n*=4 leaves could produce anomalies. Note that according to [38], 3-taxon networks could still generate anomalous gene trees. The seeming discrepancy between the two results is due to to the fact that here we define the anomaly zone in terms of all the parental trees inside the network and not just a single designated species tree.

#### **Lemma 1**

A phylogenetic network *ψ* on 3 taxa does not produce anomalies.

#### *Proof*

Let *ψ* be a phylogenetic networks on 3 taxa, and consider the set $\mathcal {W}(\psi)$ when restricted only to the distinct topologies. We have $1 \leq |\mathcal {W}(\psi)| \leq 3$.

If $|\mathcal {W}(\psi)|=3$, then the topology of every gene tree on the same set of 3 taxa is an element of $\mathcal {W}(\psi)$. Therefore, no gene tree can satisfy Eq. (1).

If $|\mathcal {W}(\psi)|=2$, without loss of generality, let the two parental trees be ((*A*,*B*),*C*) and (*A*,(*B*,*C*)). If *ψ* produces an anomaly, then it must be that the anomalous gene tree is ((*a*,*c*),*b*). To obtain this gene tree, *a* and *c* must coalesce above the root in both parental trees. Since for the other two gene trees the coalescence events could occur under or above the root, the probability of each of them is bounded from below by the probability of ((*a*,*c*),*b*). Therefore, ((*a*,*c*),*b*) is not anomalous.

If $|\mathcal {W}(\psi)| = 1$, without loss of generality, let the parental tree topology be ((*A*,*B*),*C*). If *ψ* produces an anomalous gene tree, then it must be that the anomalous gene tree is either ((*a*,*c*),*b*) or (*a*,(*b*,*c*)). To obtain ((*a*,*c*),*b*),*a* and *c* must coalesce above the root in the parental tree. And to obtain (*a*,(*b*,*c*)),*b* and *c* must also coalesce above the root in the parental tree. Since for ((*a*,*b*),*c*) the coalescence events could occur under or above the root, its probability is bounded from below by the maximum of the probabilities of ((*a*,*c*),*b*) and (*a*,(*b*,*c*)). Therefore neither ((*a*,*c*),*b*) nor (*a*,(*b*,*c*)) is anomalous. □

**Fig. 4:**
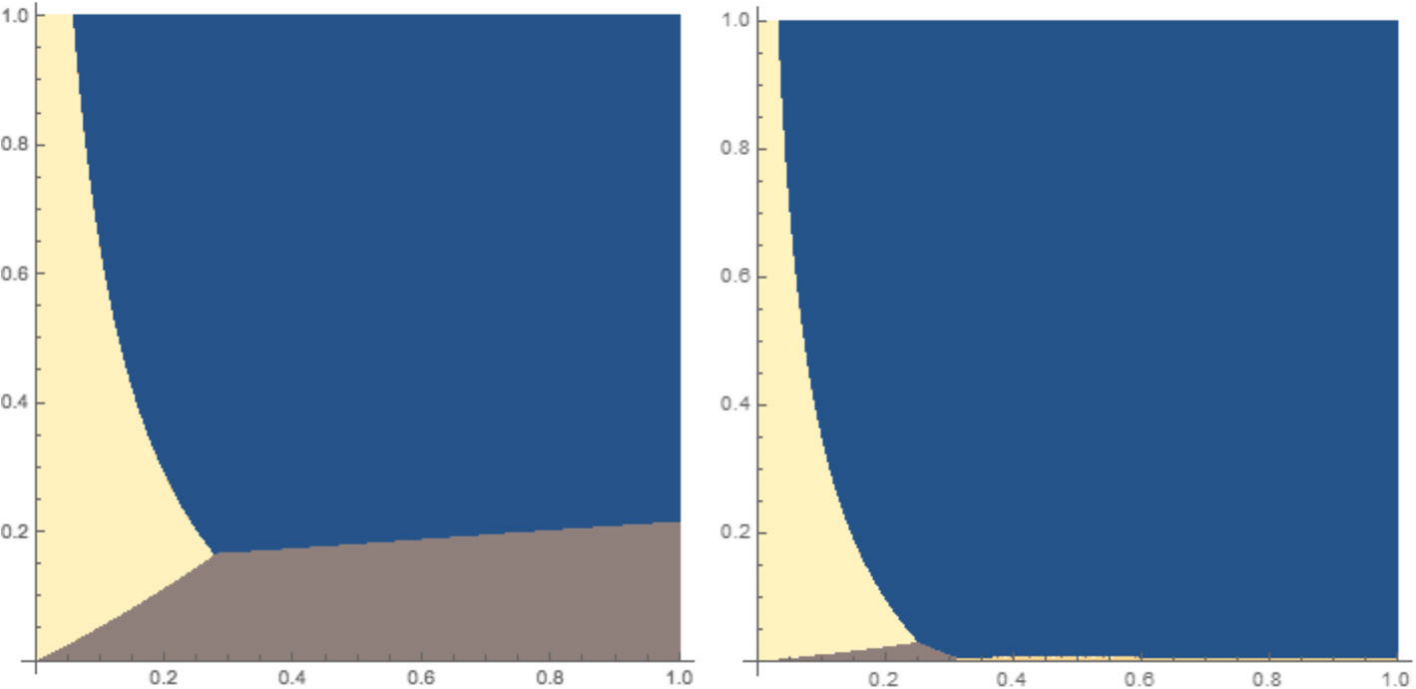
The most likely gene tree given the phylogenetic network in Fig. 2 b (with *w*=0) with *γ*=0.5 (*left*) and *γ*=0.05 (*right*). The *x*-axis corresponds to branch length *x* and the *y*-axis corresponds to branch length *y*. *Yellow*: gene tree ((*a*,*d*),(*b*,*c*)); *Orange*: gene trees (*a*,(*b*,(*c*,*d*))) and (*a*,(*c*,(*b*,*d*))); *Brown*: gene trees ((*a*,*b*),(*c*,*d*)) and ((*a*,*c*),(*b*,*d*)); *Blue*: gene tree (*a*,((*b*,*c*),*d*)) in both panels, and gene tree ((*a*,(*b*,*c*)),*d*) additionally in the *left* panel

Consider now the symmetric phylogenetic network *ψ* in Fig. 2 b and whose set of parental trees in given in Fig. 3. The four gene trees that are identical to the parental trees of the network are ((*a*,(*b*,*c*)),*d*),(*a*,((*b*,*c*),*d*)),((*a*,*b*),(*c*,*d*)) and ((*a*,*c*),(*b*,*d*)). We plotted in Fig. 4 the anomaly zone for this network in terms of small values for *x* and *y* (≤1.0) and for two values of the inheritance probability *γ*. The yellow and orange regions correspond to the anomaly zone of this network. This figure clearly shows the existence of an anomaly zone of the network in Fig. 2 b (where *w* is set to 0), which means that symmetric phylogenetic networks with *n*=4 leaves could produce anomalies.

### On the backbone tree of a phylogenetic network

A very important question in the area of phylogenetic network inference is whether there exists a tree that can be augmented into the network by adding reticulation edges between pairs of the tree’s edges. Here, we refer to such a tree as the network’s *backbone tree*. A biological significance of this tree lies in its potential designation as the species tree (e.g., see the species tree underlying the phylogenetic network of mosquitos in [44]).

Francis and Steel [35] recently introduced the notion of *tree-based networks* to capture those networks that can be obtained by augmenting a backbone tree (they called it the “base tree”). Zhang [45] and Jetten and van Iersel [46] provided necessary and sufficient conditions for tree-based networks.

The blue regions in the two panels of Fig. 4 correspond to the parameter zones where the most likely gene tree is one of the two backbone trees. However, the other regions correspond to parameter zones where the most likely gene tree is not a backbone of the network. We now provide more details on this issue.

Let us consider again the network of Fig. 2 b. This network is tree-based and each of the two trees in Fig. 1 could serve as its backbone (indeed, the same network is drawn in Fig. 1 in a way that clearly demonstrates that it is tree-based). The probabilities of all 15 gene trees under this phylogenetic network are given in Table 1.

**Table 1:** Probabilities of 15 rooted gene trees given the phylogenetic network *ψ* of Fig. 2 b (*w*=0). The quantity *g*_*ij*_(*t*) is the probability that *i* lineages coalesce into *j* lineages within time *t* [36]

Gene Tree *T*_*i*_	*P*(*T*_*i*_|*ψ*,*x*,*y*,*γ*)
*T*_1_=(((*b*,*c*),*a*),*d*)	$g_{21}(y)[\gamma (g_{21}(x)+g_{22}(x)\frac {1}{3})+(1-\gamma)(g_{22}(x)\frac {1}{3})]$
	$+g_{22}(y)[\gamma ^{2}(g_{31}(x)\frac {1}{3}+g_{32}(x)\frac {1}{3}\frac {1}{3}+g_{33}(x)\frac {1}{6}\frac {1}{3})$
	$+(1-\gamma)^{2}(g_{32}(x)\frac {1}{3}\frac {1}{3}+g_{33}(x)\frac {1}{6}\frac {1}{3})$
	$+2\gamma (1-\gamma)(g_{22}(x)g_{22}(x)\frac {1}{6}\frac {1}{3})]$
*T*_2_=(((*b*,*c*),*d*),*a*)	$g_{21}(y)[(1-\gamma)(g_{21}(x)+g_{22}(x)\frac {1}{3})+\gamma (g_{22}(x)\frac {1}{3})]$
	$+g_{22}(y)[(1-\gamma)^{2}(g_{31}(x)\frac {1}{3}+g_{32}(x)\frac {1}{3}\frac {1}{3}+g_{33}(x)\frac {1}{6}\frac {1}{3})$
	$+\gamma ^{2}(g_{32}(x)\frac {1}{3}\frac {1}{3}+g_{33}(x)\frac {1}{6}\frac {1}{3})$
	$+2\gamma (1-\gamma)(g_{22}(x)g_{22}(x)\frac {1}{6}\frac {1}{3})]$
*T*_3_=((*a*,*b*),(*c*,*d*))	$g_{22}(y)[(\gamma ^{2}+(1-\gamma)^{2})(g_{32}(x)\frac {1}{3}\frac {1}{3}+g_{33}(x)\frac {2}{6}\frac {1}{3})$
	$+\gamma (1-\gamma)(g_{21}(x)g_{21}(x)+g_{21}(x)g_{22}(x)\frac {1}{3}+g_{22}(x)g_{21}(x)\frac {1}{3}+g_{22}(x)g_{22}(x)\frac {2}{6}\frac {1}{3})$
	$+\gamma (1-\gamma)(g_{22}(x)g_{22}(x)\frac {2}{6}\frac {1}{3})]$
*T*_4_=((*a*,*c*),(*b*,*d*))	$g_{22}(y)[(\gamma ^{2}+(1-\gamma)^{2})(g_{32}(x)\frac {1}{3}\frac {1}{3}+g_{33}(x)\frac {2}{6}\frac {1}{3})$
	$+\gamma (1-\gamma)(g_{21}(x)g_{21}(x)+g_{21}(x)g_{22}(x)\frac {1}{3}+g_{22}(x)g_{21}(x)\frac {1}{3}+g_{22}(x)g_{22}(x)\frac {2}{6}\frac {1}{3})$
	$+\gamma (1-\gamma)(g_{22}(x)g_{22}(x)\frac {2}{6}\frac {1}{3})]$
*T*_5_=(((*a*,*b*),*c*),*d*)	$g_{22}(y)[\gamma ^{2}(g_{31}(x)\frac {1}{3}+g_{32}(x)\frac {1}{3}\frac {1}{3}+g_{33}(x)\frac {1}{6}\frac {1}{3})+(1-\gamma)^{2}(g_{33}(x)\frac {1}{6}\frac {1}{3})$
	$+\gamma (1-\gamma)(g_{21}(x)g_{22}(x)\frac {1}{3}+g_{22}(x)g_{22}(x)\frac {1}{6}\frac {1}{3})+\gamma (1-\gamma)g_{22}(x)g_{22}(x)\frac {1}{6}\frac {1}{3}]$
*T*_6_=(((*a*,*c*),*b*),*d*)	$g_{22}(y)[\gamma ^{2}(g_{31}(x)\frac {1}{3}+g_{32}(x)\frac {1}{3}\frac {1}{3}+g_{33}(x)\frac {1}{6}\frac {1}{3})+(1-\gamma)^{2}(g_{33}(x)\frac {1}{6}\frac {1}{3})$
	$+\gamma (1-\gamma)(g_{21}(x)g_{22}(x)\frac {1}{3}+g_{22}(x)g_{22}(x)\frac {1}{6}\frac {1}{3})+\gamma (1-\gamma)g_{22}(x)g_{22}(x)\frac {1}{6}\frac {1}{3}]$
*T*_7_=(*a*,(*b*,(*c*,*d*)))	$g_{22}(y)[(1-\gamma)^{2}(g_{31}(x)\frac {1}{3}+g_{32}(x)\frac {1}{3}\frac {1}{3}+g_{33}(x)\frac {1}{6}\frac {1}{3})+\gamma ^{2}(g_{33}(x)\frac {1}{6}\frac {1}{3})$
	$+\gamma (1-\gamma)(g_{21}(x)g_{22}(x)\frac {1}{3}+g_{22}(x)g_{22}(x)\frac {1}{6}\frac {1}{3})+\gamma (1-\gamma)g_{22}(x)g_{22}(x)\frac {1}{6}\frac {1}{3}]$
*T*_8_=(((*b*,*d*),*c*),*a*)	$g_{22}(y)[(1-\gamma)^{2}(g_{31}(x)\frac {1}{3}+g_{32}(x)\frac {1}{3}\frac {1}{3}+g_{33}(x)\frac {1}{6}\frac {1}{3})+\gamma ^{2}(g_{33}(x)\frac {1}{6}\frac {1}{3})$
	$+\gamma (1-\gamma)(g_{21}(x)g_{22}(x)\frac {1}{3}+g_{22}(x)g_{22}(x)\frac {1}{6}\frac {1}{3})+\gamma (1-\gamma)g_{22}(x)g_{22}(x)\frac {1}{6}\frac {1}{3}]$
*T*_9_=((*a*,*d*),(*b*,*c*))	$g_{21}(y)[\gamma g_{22}(x)\frac {1}{3}+(1-\gamma)g_{22}(x)\frac {1}{3}]$
	$+g_{22}(y)[\gamma ^{2}(g_{32}(x)\frac {1}{3}\frac {1}{3}+g_{33}(x)\frac {2}{6}\frac {1}{3})+(1-\gamma)^{2}(g_{32}(x)\frac {1}{3}\frac {1}{3}+g_{33}(x)\frac {2}{6}\frac {1}{3})$
	$+\gamma (1-\gamma)(g_{22}(x)g_{22}(x)\frac {2}{6}\frac {1}{3})+\gamma (1-\gamma)(g_{22}(x)g_{22}(x)\frac {2}{6}\frac {1}{3})]$
*T*_10_=(((*a*,*b*),*d*),*c*)	$g_{22}(y)[\gamma ^{2}(g_{32}(x)\frac {1}{3}\frac {1}{3}+g_{33}(x)\frac {1}{6}\frac {1}{3})+(1-\gamma)^{2}(g_{33}(x)\frac {1}{6}\frac {1}{3})$
	$+\gamma (1-\gamma)(g_{21}(x)g_{22}(x)\frac {1}{3}+g_{22}(x)g_{22}(x)\frac {1}{6}\frac {1}{3})+\gamma (1-\gamma)(g_{22}(x)g_{22}(x)\frac {1}{6}\frac {1}{3})]$
*T*_11_=(*b*,(*a*,(*c*,*d*)))	$g_{22}(y)[(1-\gamma)^{2}(g_{32}(x)\frac {1}{3}\frac {1}{3}+g_{33}(x)\frac {1}{6}\frac {1}{3})+\gamma ^{2}(g_{33}(x)\frac {1}{6}\frac {1}{3})$
	$+\gamma (1-\gamma)(g_{21}(x)g_{22}(x)\frac {1}{3}+g_{22}(x)g_{22}(x)\frac {1}{6}\frac {1}{3})+\gamma (1-\gamma)(g_{22}(x)g_{22}(x)\frac {1}{6}\frac {1}{3})]$
*T*_12_=(((*a*,*d*),*b*),*c*)	$g_{22}(y)[\gamma ^{2}(g_{33}(x)\frac {1}{6}\frac {1}{3})+(1-\gamma)^{2}(g_{33}(x)\frac {1}{6}\frac {1}{3})$
	$+\gamma (1-\gamma)(g_{22}(x)g_{22}(x)\frac {1}{6}\frac {1}{3})+\gamma (1-\gamma)(g_{22}(x)g_{22}(x)\frac {1}{6}\frac {1}{3})]$
*T*_13_=(((*b*,*d*),*a*),*c*)	$g_{22}(y)[\gamma ^{2}(g_{33}(x)\frac {1}{6}\frac {1}{3})+(1-\gamma)^{2}(g_{32}(x)\frac {1}{3}\frac {1}{3}+g_{33}(x)\frac {1}{6}\frac {1}{3})$
	$+\gamma (1-\gamma)(g_{22}(x)g_{22}(x)\frac {1}{6}\frac {1}{3})+\gamma (1-\gamma)(g_{21}(x)g_{22}(x)\frac {1}{3}+g_{22}(x)g_{22}(x)\frac {1}{6}\frac {1}{3})]$
*T*_14_=(((*a*,*c*),*d*),*b*)	$g_{22}(y)[(1-\gamma)^{2}(g_{33}(x)\frac {1}{6}\frac {1}{3})+\gamma ^{2}(g_{32}(x)\frac {1}{3}\frac {1}{3}+g_{33}(x)\frac {1}{6}\frac {1}{3})$
	$+\gamma (1-\gamma)(g_{22}(x)g_{22}(x)\frac {1}{6}\frac {1}{3})+\gamma (1-\gamma)(g_{21}(x)g_{22}(x)\frac {1}{3}+g_{22}(x)g_{22}(x)\frac {1}{6}\frac {1}{3})]$
*T*_15_=(((*a*,*d*),*c*),*b*)	$g_{22}(y)[\gamma ^{2}(g_{33}(x)\frac {1}{6}\frac {1}{3})+(1-\gamma)^{2}(g_{33}(x)\frac {1}{6}\frac {1}{3})$
	$+\gamma (1-\gamma)(g_{22}(x)g_{22}(x)\frac {1}{6}\frac {1}{3})+\gamma (1-\gamma)(g_{22}(x)g_{22}(x)\frac {1}{6}\frac {1}{3})]$

**Fig. 5:**
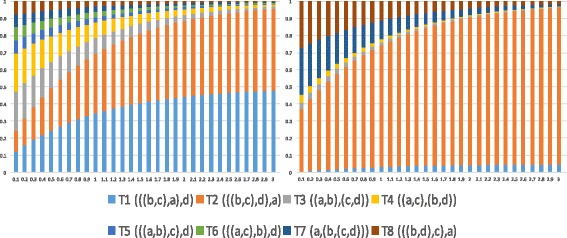
Gene tree distribution for the phylogenetic network in Fig. 2 b (*w*=0) as *x*→*∞*, for *γ*=0.5 (*left*) and *γ*=0.05 (*right*). The *x*-axis corresponds to branch length *y* and the *y*-axis corresponds to the probability of each gene tree topology (see Table 2)

While there are 15 possible gene tree topologies on taxa *a*,*b*,*c*, and *d*, as branch length *x* in the network tends to infinity, the probabilities of seven of the 15 gene tree topologies converge to 0 and only eight gene trees have non-zero mass: ((*a*,(*b*,*c*)),*d*), (*a*, ((*b*,*c*),*d*)), ((*a*,*b*), (*c*,*d*)), ((*a*,*c*),(*b*,*d*)), (((*a, b*), *c*), *d*), (((*a*, *c*), *b*), *d*), (*a*, (*b*, (*c*, *d*))), and (*a*, (*c*, (*b*, *d*))). The probabilities in this case are given in Table 2 and visualized as a function of varying branch length *y* for two different settings of *γ* in Fig. 5.

**Table 2:** Probabilities of 15 rooted gene trees given the phylogenetic network *ψ* of Fig. 2 b (*w*=0) as *x*→*∞*

Gene Tree *T*_*i*_	*P*(*T*_*i*_|*ψ*,*y*,*γ*)
*T*_1_=(((*b*,*c*),*a*),*d*)	$\gamma -(\gamma -\frac {\gamma ^{2}}{3})e^{-y}$
*T*_2_=(((*b*,*c*),*d*),*a*)	$(1-\gamma)-(-\frac {\gamma ^{2}}{3}-\frac {\gamma }{3}+\frac {2}{3})e^{-y}$
*T*_3_=((*a*,*b*),(*c*,*d*))	*γ*(1−*γ*)*e* ^−*y*^
*T*_4_=((*a*,*c*),(*b*,*d*))	*γ*(1−*γ*)*e* ^−*y*^
*T*_5_=(((*a*,*b*),*c*),*d*)	$\frac {\gamma ^{2}}{3}e^{-y}$
*T*_6_=(((*a*,*c*),*b*),*d*)	$\frac {\gamma ^{2}}{3}e^{-y}$
*T*_7_=(*a*,(*b*,(*c*,*d*)))	$\frac {(1-\gamma)^{2}}{3}e^{-y}$
*T*_8_=(((*b*,*d*),*c*),*a*)	$\frac {(1-\gamma)^{2}}{3}e^{-y}$
*T*_9_=((*a*,*d*),(*b*,*c*))	0
*T*_10_=(((*a*,*b*),*d*),*c*)	0
*T*_11_=(*b*,(*a*,(*c*,*d*)))	0
*T*_12_=(((*a*,*d*),*b*),*c*)	0
*T*_13_=(((*b*,*d*),*a*),*c*)	0
*T*_14_=(((*a*,*c*),*d*),*b*)	0
*T*_15_=(((*a*,*d*),*c*),*b*)	0

When *γ*=0.5 and $\frac {1}{4}e^{-y}>\frac {1}{2}-\frac {5}{12}e^{-y}$, which is equivalent to *y*<0.288, the most likely gene tree given *ψ* is not one of its backbone trees (that is, the network cannot be obtained by a adding a single reticulation edge to the most likely gene tree). This also demonstrates that if we defined anomalies in terms of the set $\mathcal {U}(\psi)$ instead of set $\mathcal {W}(\psi)$, the phylogenetic network would still produce anomalous gene trees.

Given that the most likely gene tree is not necessarily a backbone of the phylogenetic network, we now turn our attention to three recent methods whose goal is to infer a species tree despite horizontal gene transfer. It is very important to point out upfront that the assumptions of these methods do not necessarily match the scenarios we investigate here, but our goal is to assess how well they do at recovering a backbone tree inside the network of Fig. 2 b. In [34], Davidson et al. showed that ASTRAL-II [47] performed best among species tree inference methods in terms of recovering the species tree in the presence of reticulation (under a specific model of horizontal gene transfer). They further proved that the method is statistically consistent in terms of recovering the species tree under the same model. In [32], Steel et al. showed that triplet-based approaches to species tree inference are consistent in terms of inferring a species tree in the presence of horizontal gene transfer (also under a specific model). This technique was implemented as the “primordial tree” in Dendroscope [48]. Both ASTRAL-II and the primordial tree method in Dendroscope take gene trees as input. The method of Daskalakis and Roch [33] takes as input gene trees with branch length and compute the distance between every two taxa *u* and *v* as the median of the gene-tree distances between *u* and *v* over all gene trees in the data set (given a gene tree with branch lengths, the gene-tree distance between two leaves is the sum of the branch lengths on the simple path between the two leaves).

**Fig. 6:**
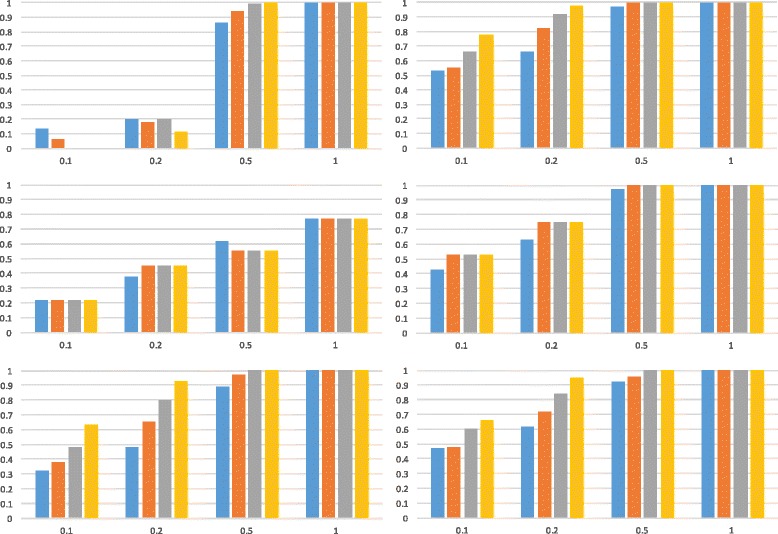
The accuracy of three methods for inferring species trees in the presence of reticulation on data generated on the phylogenetic network of Fig. 2 b. *Left* column corresponds to setting *γ*=0.5 and *right* column corresponds to setting *γ*=0.05. Four settings for branch length *y* (on the *x*-axis) were used, and for each setting data sets with 25, 50, 100, and 200 loci were generated. See the text for definition of the accuracy measure. (*Top*) ASTRAL-II [47]; (*Middle*) The method of Steel et al. [32] as implemented in Dendroscope [48]; (*Bottom*) Our own implementation of the method of Daskalakis and Roch [33]. The four bars for each setting of *y* correspond from *left* to *right* to 25, 50, 100, and 200 loci, respectively

We simulated gene tree data sets under the phylogenetic network of Fig. 2 b using ms [49] while varying branch length *y* to take on values from the set {0.1,0.2,0.5,1.0} (*w* was set to 0 and *x* was set to 1000 so as to rule out deep coalescence involving the two branches incident with the root). Data sets with 25,50,100 and 200 gene trees were generated, and for each configuration of branch length *y* and number of gene trees, 100 data sets were simulated. The accuracy of each method for a setting of branch length *y* and number of gene trees is the fraction, out of the 100 data sets, of times that the method returned one of the two trees displayed by the network. The results for all three methods on the simulated data are shown in Fig. 6.

The results show that when *y* is very small, the methods perform poorly in terms of returning one of the two trees displayed by the network, especially in the case of *γ*=0.5. This is expected as an inheritance probability of 0.5 is a huge deviation from the assumptions of the three methods. When *γ*=0.5 and *y* is long enough (e.g., 1), ASTRAL-II and the method of [33] do a perfect job, while the method of [32] does not perform as well. For smaller values of *y* and with *γ*=0.5, the method of [33] consistently performs better than the other two methods. For *γ*=0.05, which is closer to the assumptions of the methods, all three of them perform well, even when *y*=0.5 (in this case, the most likely gene tree is also a backbone tree). For smaller values of *y* in this case, ASTRAL-II and the method of [33] do almost equally well, and slightly better than the method of [32].

Our results are in agreement with the findings in [38], where the authors showed, additionally, that methods for phylogenetic network inference (specifically, they evaluated the maximum likelihood method of [50] in PhyloNet [42]) do a better job at recovering a species tree in the presence of gene flow than methods that infer (species) trees.

### From gene trees to species networks via parental trees: a clustering approach

Given our discussion above of the set of parental trees, one can view a phylogenetic network *ψ* as a mixture model with $|\mathcal {W}(\psi)|$ components and each component as a distribution on gene trees defined by the parental tree corresponding to that component. This view gives rise to a novel approach for reconstructing phylogenetic networks from a set $\mathcal {G}$ of gene trees when both deep coalescence and reticulation could be both at play:

1. Cluster the gene trees into clusters *C*_1_,*C*_2_,…,*C*_*k*_;
2. Infer a parental tree *T*_*i*_ for cluster *C*_*i*_ under the multispecies coalescent;
3. Combine the trees *T*_1_,*T*_2_,…,*T*_*k*_ into a phylogenetic network *ψ*.

The rationale behind this approach is that clustering would identify the components of the mixture model, where the gene trees belonging to a component differ only because of incomplete lineage sorting (ILS), but not because of hybridization. That is why in Step (2) a tree is inferred for each component under the multispecies coalescent, which only handles ILS. In the third step, disagreements among the *k* inferred trees are assumed to be all due to the hybridization events, and are used to obtain the final network. A parsimony approach, for example, to Step (3) would be formulated as follows.

#### **Definition 5**

The Parental Tree Network Problem is defined as:

- **Input:** A set ${\mathcal {P}}$ of parental trees.
- **Output:** A phylogenetic network *ψ* with the smallest number of reticulation nodes such that ${\mathcal {P}} \subseteq \mathcal {W}(\psi)$.

Establishing the computational complexity of this newly defined problem and devising algorithms and heuristics for solving it are beyond the scope of this paper.

In [39], Gori et al. studied the performance of various combinations of clustering methods and dissimilarity measures on gene tree topologies as well as gene trees with branch lengths. In our work here, the focus is on phylogenetic network inference and our simulation study in what follows is preliminary and aimed at demonstrating the viability of this approach in terms of identifying the true set of parental trees.

**Fig. 7:**
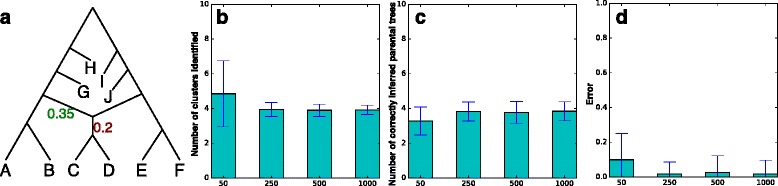
Performance of the clustering approach on the simulated data as a function of the number of gene trees. **a** The phylogenetic network used in the simulations. The lengths of the two reticulation edges were set to 0. The length of the edge going out of the reticulation node was set to 0.2. The inheritance probability of the *left* reticulation edge was set to 0.35. Ten networks were generated from this network by setting the length of each other internal branch to a random number uniformly sampled in the range [0.7,1.3]. **b** The number of clusters identified (averaged over 300 data sets for each bar). **c** The number of correctly inferred parental trees (out of the maximum of four parental trees). **d** The error between the set of inferred trees from the identified clusters and the set of four parental trees of the network. The *x*-axis in panels (**b**)-(**d**) corresponds to the number of gene trees

We used 10 phylogenetic networks (Fig. 7 a), and within each, we generated 30 data sets of 50 gene trees each, 30 data sets of 250 gene trees each, 30 data sets of 500 gene trees each, and 30 data sets of 1000 gene trees each.

For each gene tree data set, pairwise Robinson-Foulds (RF) [51] distances were computed between the gene trees, and the pairwise distances were converted into 3-dimensional points in Euclidean space using multidimensional scaling (MDS) as implemented in the MDSJ package [52] (we also conducted clustering directly on the RF distances, and found a significant improvement in the results after applying MDS). We implemented the *k*-means clustering algorithm [53] and used it to cluster the gene trees based on the Euclidean distances from MDS using *k*=2,3,…,10. We implemented the silhouette method [54] and the number of clusters with the maximum average silhouette (based on the pairwise RF distances) was selected as the number of clusters identified and the corresponding clustering as the identified clusters.

Figure 7 b shows the results of identifying the number of clusters (the correct number is 4). As the figure shows, clustering in this case is performing very well, returning the correct number of clusters in almost all cases with 250 gene trees or more, and performing only slightly poorer in the case of 50 gene trees.

After the clusters were identified, we turned to the next natural question: Do the clusters correspond to the parental trees of the network? To investigate this question, we chose to apply the “minimizing deep coalescence” (MDC) method of [55] as implemented in [50] (the heuristic version that uses only the clusters in the input gene trees) to infer a “species tree” on each cluster. We then quantified the number of true parental trees that were inferred by MDC on the clusters in each data set. The results are shown in Fig. 7 c. The results indicate a very good performance where all four true parental trees are almost always correctly inferred, particularly when 250 gene trees or more are used.

Finally, when this MDC-based analysis returns trees other than the true parental trees, how far are they from the true ones? To answer this question we compared the set of true parental trees and the set of trees inferred by MDC based on the identified clusters using the tree-based measure of [56] (finding the min-weight edge cover of a bipartite graph whose two sets of nodes correspond to these two sets of trees and the weights of edges are RF distance) as implemented in PhyloNet [50]. The results are shown in Fig. 7 d. The results indicate a very good performance of about 2 % error for data sets with 250 gene trees or more, and about 10 % for data sets with 50 gene trees.

It is worth mentioning that if a network that displays all gene trees in the input was sought, the result would be a network that differs significantly from the true network, as each data set contained many distinct gene tree topologies. All the differences among gene trees (many of which are due to ILS) would be interpreted as signal for reticulations. This highlights the major difference between the current practice of seeking a network that displays all gene trees in the input and our proposed approach of seeking a network whose parental trees are obtained from the input gene trees.

## Conclusions

In this paper, we showed that when deep coalescence occurs, inference and analysis of phylogenetic networks are more adequately done with respect to the set of parental trees of the network, rather than the common practice of using the set of trees displayed by the network. We described the simple procedure for enumerating the set of parental trees of a given network, and based on this set, we made three contributions. First, we defined the anomaly zone for a phylogenetic network topology as the region of branch lengths and inheritance probabilities under which the most likely gene tree is not one of the parental trees inside the network. We provided straightforward results on the anomaly zones for networks that mainly result from the fact that networks are an extension of trees. An important question is whether it is feasible that none of the trees displayed by a network has an anomaly zone, yet the network itself has one.

In many cases, biologists are interested in identifying a species tree in the presence of gene flow. We demonstrated that in the presence of deep coalescence, the most likely gene tree is not necessarily one of the backbone trees inside the network. Furthermore, we studied the performance of three recently introduced methods in terms of their ability to recover a backbone tree inside the network. We found that none of these methods performs well when deep coalescence is extensive. It is important to point out, though, that none of these methods were designed specifically for cases of hybridization, where multiple genomic loci could be introgressed due to the same hybridization event. However, our findings here call for more research into the question of identifying a species tree inside the network, when one exists. However, biologically, reticulation could be extensive, such as reported recently in an analysis of a mosquito data set [44, 57], in which case, designating a “species tree” might not be adequate [58]. Furthermore, as Solís-Lemus et al. [38] showed, inferring a network does a better job at finding even the species tree when gene flow is at play. From a computational perspective, identifying such a tree aids significantly in searching for networks from data [42, 59] as they can serve as the starting phylogeny to which reticulation edges could be added.

Finally, many existing approaches for network inference rely on the assumption that the input gene trees are a subset of the set of trees displayed by a network and, consequently, seek to infer a phylogenetic network that displays all the gene trees. In the presence of deep coalescence, this approach would result in very erroneous networks. We argued that in this situation, parental trees need to be inferred first from gene trees and then a network that contains the inferred parental trees could be estimated. To demonstrate the merit for this approach, we introduced a method by which gene trees are first clustered and then parental trees are inferred for the clusters. The results were very promising for this clustering-based approach to be pursued further. In terms of network inference, this approach gives rise to a new computational problem in which a network is sought to contain a given set of parental trees. It is important to acknowledge here that our performance study of the clustering approach is very preliminary and is aimed at introducing the problem and demonstrating its merit in a relatively ideal setting. We identify as a direction for future research a thorough analysis that examines, among many other aspects, the effects of errors in gene tree estimates (as opposed to using true gene trees), larger variations in the network’s branch lengths, and the number of reticulations in the network, on the performance of the approach.
